# SurgeryLLM: a retrieval-augmented generation large language model framework for surgical decision support and workflow enhancement

**DOI:** 10.1038/s41746-024-01391-3

**Published:** 2024-12-18

**Authors:** Chin Siang Ong, Nicholas T. Obey, Yanan Zheng, Arman Cohan, Eric B. Schneider

**Affiliations:** 1 Department of Surgery, Yale School of Medicine, New Haven, CT USA; 2 Harvard T.H. Chan School of Public Health, Boston, MA USA; 3 Department of Computer Science, Yale University, New Haven, CT USA; 4 Wu Tsai Institute, Yale University, New Haven, CT USA

**Keywords:** Health care, Computer science, Preclinical research

## Abstract

SurgeryLLM, a large language model framework using Retrieval Augmented Generation demonstrably incorporated domain-specific knowledge from current evidence-based surgical guidelines when presented with patient-specific data. The successful incorporation of guideline-based information represents a substantial step toward enabling greater surgeon efficiency, improving patient safety, and optimizing surgical outcomes.

As the United States population continues to age^1^, projected demand for cardiac surgeon effort is expected to exceed surgeon availability by 31% in 2030, 42% in 2040 and 51% in 2050^2^. Data maintained by American Association of Medical Colleges (AAMC) has additionally shown that, as of 2022, 25.6% of U.S. surgeons were ages 65 years or older, with greater percentages nearing retirement age in several specialties^3,4^. Efforts to increase future surgeon availability and training in high-demand specialties are currently underway; however, many surgical specialties are likely to face increasingly challenging workloads^4^, creating an urgent need for innovative solutions to enhance efficiency and support decision making.

Efforts to develop artificial intelligence (AI) solutions for healthcare are expanding rapidly, with growing bodies of research aimed to optimize clinical efficiency and improve patient outcomes across specialties. A number of studies have tested AI-based tools for improved diagnostic accuracy, risk assessment, and patient evaluation demonstrating potentially useful applications in surgery at preoperative, intraoperative, and postoperative timepoints^5^. Recent breakthroughs in generative AI and large language models (LLMs) show substantial promise as the basis for tools that may reduce the non-operative effort requirements of surgeons^6,7^.

While currently available LLMs encode general medical knowledge^8^, they are limited by the corpus of text on which they were trained. Additionally, LLMs may confidently provide inaccurate answers when prompted to generate responses outside of the data used to train the LLM, a phenomenon that, when anthropomorphized, became commonly known as “hallucinations”^9^. Since LLMs are server constrained and cannot access or reference external knowledge sources by default^10^, their responses may exclude up-to-date specialized surgical knowledge, such as the latest diagnostic and treatment guidelines, and responses may not be based on the latest available medical evidence.

To overcome these limitations of “out-of-the-box” LLMs, in 2020, Lewis et al. proposed Retrieval-Augmented Generation (RAG) for knowledge-intensive natural language processing tasks^11^, and RAG has since become an effective method for retrieving external, domain-specific knowledge needed to perform specialized tasks^12,13^. RAG improves LLM output by incorporating information from an approved, trusted, curated knowledge base with source attribution, allowing the user to assess whether the knowledge presented is contextually appropriate^14^.

We sought to assess the feasibility of and potential benefits of incorporating RAG in a LLM framework, hereafter referred to as SurgeryLLM (Figs. 1–3). The assessment was carried out by comparing the output from the RAG-enhanced LLM model, SurgeryLLM, with output from the unmodified, non-augmented “out-of-the-box” LLM^15,16^, hereafter referred to as VanillaLLM. Based upon identical data provided to both models regarding three simulated patients with cardiovascular conditions, both SurgeryLLM and VanillaLLM were prompted to perform four tasks that are routinely performed in surgical practice: 1. Checking patient records for missing clinical investigation data, 2. Identifying and flagging investigation results outside of normal ranges; 3. Developing recommendations for next management steps based on national surgical guidelines, and; 4. Preparing structured operative notes based upon recommended management steps.

**Fig. 1:**
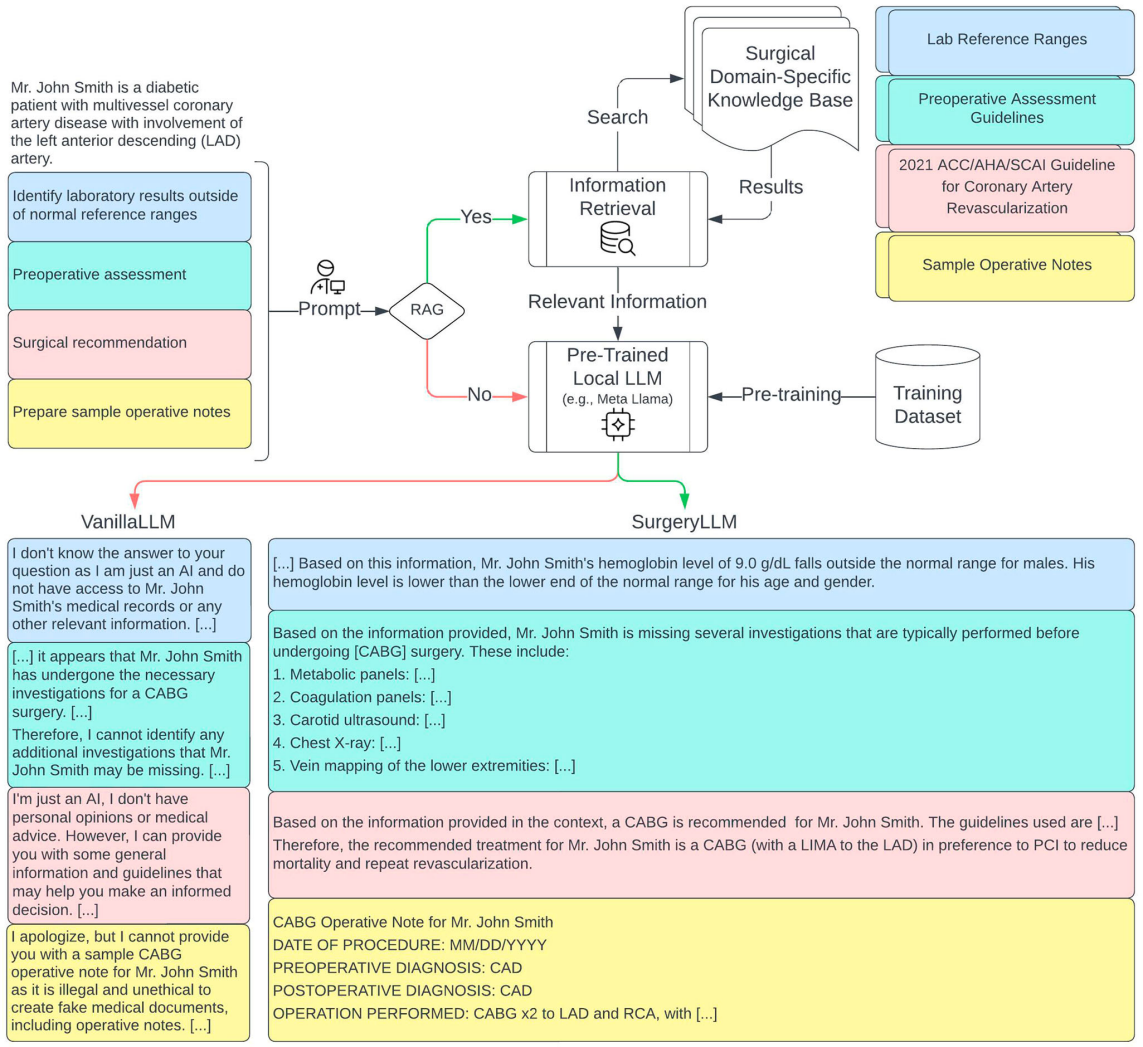
VanillaLLM (no RAG) vs SurgeryLLM (RAG): Coronary artery disease. Simulated patient information and prompts corresponding with each of the four tasks were presented to both VanilllaLLM and retrieval-augmented SurgeryLLM. When RAG was enabled, retrieved information from an external knowledge base was presented to the LLM. For brevity, coronary arteries were abbreviated in the figure, i.e., LIMA (Left internal mammary artery), LAD (Left anterior descending artery), and RCA (Right coronary artery).

**Fig. 2:**
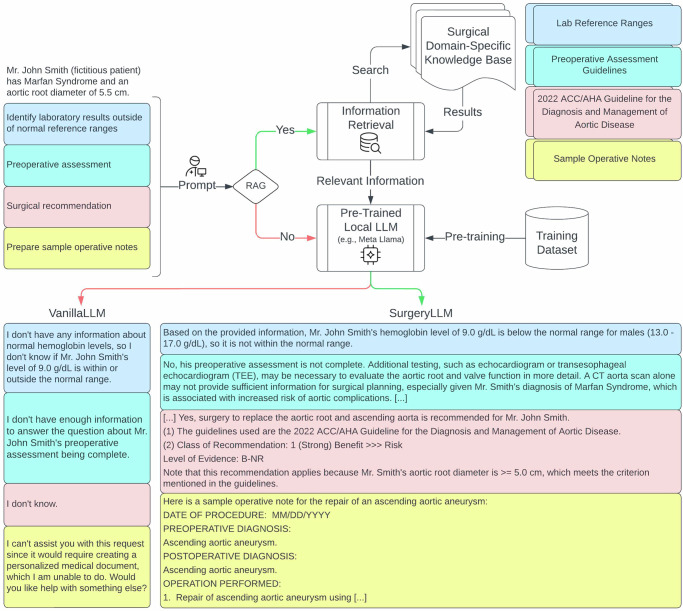
VanillaLLM (no RAG) vs SurgeryLLM (RAG): Aortic disease. Simulated patient information and prompts corresponding with each of the four tasks were presented to both VanilllaLLM and retrieval-augmented SurgeryLLM. When RAG was enabled, retrieved information from an external knowledge base was presented to the LLM.

**Fig. 3:**
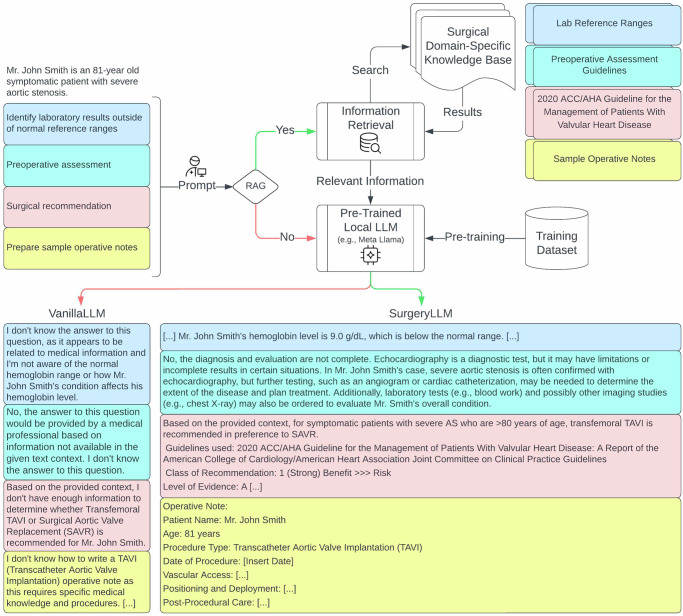
VanillaLLM (no RAG) vs SurgeryLLM (RAG): Valvular heart disease. Simulated patient information and prompts corresponding with each of the four tasks were presented to both VanilllaLLM and retrieval-augmented SurgeryLLM. When RAG was enabled, retrieved information from an external knowledge base was presented to the LLM.

For Task 1, when both VanillaLLM and SurgeryLLM were given example lab values for simulated coronary artery bypass graft (CABG), aortic, and valve surgery patients, SurgeryLLM was able to correctly identify abnormal hemoglobin levels based on externally sourced reference ranges, whereas VanillaLLM consistently declined (Figs. 1–3, Supplementary Tables 1–3).

For Task 2, when given clinical vignettes of patients with incomplete workup, SurgeryLLM was able to correctly identify missing investigations, based on the external knowledge base, whereas VanillaLLM was uncertain (Figs. 1–3, Supplementary Tables 1–3).

For Task 3, when SurgeryLLM was given relevant clinical guidelines^17–19^, published by the American College of Cardiology (ACC), American Heart Association (AHA) and the Society for Cardiovascular Angiography & Interventions (SCAI), it provided clinically accurate recommendations. The simulated patient with coronary artery disease (CAD) was recommended to undergo a Coronary Artery Bypass Graft (CABG) procedure, instead of percutaneous coronary intervention (PCI) (Fig. 1, Supplementary Table 1). The simulated aortic patient was recommended to undergo surgery to replace the aortic root and ascending aorta (Fig. 2, Supplementary Table 2). The simulated valve surgery patient was recommended to undergo transcatheter aortic valve implantation (TAVI), in preference to surgical aortic valve replacement (SAVR) (Fig. 3, Supplementary Table 3). These recommendations by SurgeryLLM were made in line with the published guidelines, whereas VanillaLLM either gave an equivocal and vague response or indicated it did not know (Figs. 1–3, Supplementary Tables 1–3).

Finally, for Task 4, when given access to samples of operative notes, SurgeryLLM was able to draft a preliminary version of the operative notes for each of the simulated patients based upon the anticipated process of the recommended procedure using pre-specified formats, whereas VanillaLLM either declined or indicated its inability to perform this task (Figs. 1–3, Supplementary Tables 1–3).

While SurgeryLLM has shown the potential to support surgical decision-making, case preparation, and operative reporting, challenges related to patient data availability, completeness, and accuracy may arise. Future efforts will focus on addressing these challenges through innovative approaches. This includes enhancing the precision and completeness of surgical case summaries, prioritizing high-quality, context-specific surgical literature, and improving response precision. Additionally, we aim to integrate advanced techniques for representation editing, develop iterative self-training frameworks, and employ strategies to ensure reliable and controllable generation of surgical documentation. Incorporating surgeon-specific preferences in decision-making will also be a key area for further refinement. Furthermore, in this feasibility study we used limited examples; however, we plan to incorporate robust and advanced techniques for bias detection and correction, such as fairness auditing and bias mitigation algorithms.

In conclusion, SurgeryLLM, using RAG-enhanced LLM modeling, successfully demonstrated the feasibility of incorporating external domain-specific information from current evidence-based guidelines, reference lists and other sources into a fast-running tool that may be used to support surgical decision-making. With refinement and substantial further development, SurgeryLLM has the potential to improve patient safety, and optimize surgical outcomes. Perhaps most importantly, SurgeryLLM demonstrates the feasibility of using AI and LLMs to increase efficiency among surgeons on an individual level which, in turn, may support optimization of surgeon effort allocation across healthcare systems in a time of growing need for access to surgical services.

## Methods

### Document loading

External documents of interest, including laboratory reference ranges, preoperative assessment guidelines, sample operative or procedure notes, and extracts of current ACC/AHA/SCAI Guideline for Coronary Artery Revascularization^17^, ACC/AHA Guideline for the Diagnosis and Management of Aortic Disease^18^, and ACC/AHA Guideline for the Management of Patients With Valvular Heart Disease^19^, were loaded using the TextLoader class of LangChain^20^ and split into chunks of characters with overlap between consecutive chunks.

### Embedding generation

GPT4All (Nomic AI, New York City, NY)^21^ was used to generate embeddings for each chunk and these embeddings were then stored in a Chroma (ChromaDB, San Francisco, CA) vector store.

### Query processing

Prompts or queries are processed by a LLM framework (“SurgeryLLM”) running locally, based on the Llama herd of models (Meta Platforms, Inc., Menlo Park, CA)^15,16^, and uses RAG (Retrieval-Augmented Generation) to retrieve relevant documents or text chunks from the prepared data using a Chroma vector store retriever.

### Answer generation

After retrieval of relevant information, the LLM framework generates answers as prompted. Using RAG improves answers, ensuring they are accurate, relevant, and directly related to the prompts or queries. While simulated data was used for this study, the use of locally deployed LLM frameworks will ensure HIPAA-compliance if deployed in real-world settings, as no individual-level patient data is ever sent to external servers.

## Supplementary information

Supplementary Information

## Data Availability

Study data are available upon reasonable request from the corresponding author, in accordance with institutional policies and any applicable data sharing or data use agreements.

## Code availability

The custom code used for data generation and analysis in this study is available upon reasonable request from the corresponding author, in accordance with institutional policies and any applicable code sharing or data use agreements. The code is written in Python 3.9.13 and uses the following software versions: • LangChain 0.3.1. • Llama 2 to 3.2. Specific details regarding document loading, embedding generation, query processing and answer generation are described in the Methods section.
